# A Green Self-Assembled Nanoplatform of 10-Hydroxycamptothecin and *Cordyceps* Polysaccharides for Dual Anti-Tumor Efficacy Through Apoptosis and Immune Modulation

**DOI:** 10.3390/pharmaceutics18030366

**Published:** 2026-03-15

**Authors:** Shu Zhou, Chunyu Zhao, Lina Sun, Huahua Li, Mengting Xv, Yikun Wang, Lihong Wang, Yining Zhang, Xinying Lu, Wenyi Huang, Yanjie Guo, Jie Bai

**Affiliations:** School of Chinese Materia Medica, Beijing University of Chinese Medicine, Beijing 100029, China; zhoushubucm@163.com (S.Z.); zhaochunyu0627@163.com (C.Z.); lina980925@163.com (L.S.); lihuahua20160907@163.com (H.L.); xmengting1850@163.com (M.X.); w2499162867@163.com (Y.W.); 18242099756@163.com (L.W.); 15578318710@163.com (Y.Z.); luxinying1999@163.com (X.L.); 18857712219@163.com (W.H.);

**Keywords:** self-assembly, molecular dynamics simulations, melanoma, dual antitumor efficacy

## Abstract

**Background:** Melanoma is one of the most dangerous types of skin cancer, with its global incidence having surged in recent years. There exists an urgent clinical need for novel therapeutic strategies that combine high efficacy, low toxicity, and multiple mechanisms of action. **Methods:** This study applies a “Property Optimization and Therapeutic Synergy” strategy, selecting the natural active polysaccharide component, *Cordyceps* polysaccharides (WCP), as a functional carrier to encapsulate the broad-spectrum chemotherapeutic agent, 10-Hydroxycamptothecin (10HCPT, HCPT). Leveraging non-covalent interactions between the two components, a self-assembly nanoscale drug delivery system (H-W NPs) with high stability and dual antitumor activity was constructed to achieve more efficient and precise antitumor effects. **Results:** The H-W NPs demonstrated outstanding antitumor efficacy both in vitro and in vivo. The H-W NPs achieved a threefold increase in the inhibition rate against B16-F10 cells compared to free HCPT in vitro and demonstrated a remarkable tumor inhibition rate of 95.08% in vivo. The therapeutic effect may be attributed to the dual antitumor mechanisms of the H-W NPs. Mechanistic studies revealed a synergistic dual-mode of action driving this potent efficacy. Firstly, H-W NPs efficiently induced caspase-3-mediated apoptosis in tumor cells. RNA sequencing analysis suggested the involvement of pathways related to cell cycle arrest and apoptosis. Additionally, H-W NPs promoted the expansion and activation of CD8^+^ T cells in the spleen. These activated cytotoxic T cells reinforced the apoptotic cascade, effectively amplifying the caspase-3-mediated death signal. **Conclusions:** In summary, the self-assembly nanoscale drug system achieved potent antitumor efficacy through the synergistic action of direct tumor cell killing and immune modulation, offering a highly promising strategy for the development of novel formulations against melanoma.

## 1. Introduction

Melanoma is a highly invasive and life-threatening skin cancer arising from the malignant transformation of melanocytes. It is among the most aggressive skin cancers, and its incidence has risen steadily worldwide in recent decades [1,2]. Surgical excision remains the primary treatment for early-stage disease, whereas chemotherapy is still the mainstay for advanced or metastatic melanoma [3,4,5]. However, conventional chemotherapeutics lack tumor selectivity, penetrate poorly into tumor tissue, and cause considerable systemic toxicity, all of which limit their clinical utility [6]. Consequently, there is an urgent need for novel anti-melanoma agents that combine high efficacy, low toxicity, and multiple mechanisms of action. To address the inherent limitations of traditional chemotherapy therapy’s low efficacy and high toxicity, nanoscale drug delivery systems have become a major focus of research because of their reduced side-effects, appropriate half-life, high bioavailability, and capacity for targeted delivery [7,8,9,10].

10-Hydroxycamptothecin is a broad-spectrum antitumor agent with enhanced efficacy and reduced toxicity, demonstrating significant antitumor activity in liver, breast, lung, ovarian, and other solid tumors [11,12,13]. HCPT selectively inhibits DNA topoisomerase I during the S phase, suppressing cleaved DNA strand degradation and inducing apoptosis [14,15,16,17]. Since HCPT exhibits no cross-resistance with commonly used clinical antitumor agents, it is frequently combined with other agents to enhance therapeutic efficacy. However, clinical application of HCPT is limited by its poor water solubility, unstable lactone ring structure, and low bioavailability. Polymeric materials are frequently used to improve the solubility of hydrophobic chemotherapeutic agents, thereby facilitating drug delivery and synergistic therapy [18,19,20,21,22]. However, carrier materials may be associated with limitations, such as low drug-loading capacity and increased metabolic burden [23]. To overcome this challenge, naturally derived, low-toxicity bioactive compounds are co-assembled with antitumor drugs to construct carrier-free nanostructures [24]. This strategy not only circumvents the inherent limitations of carrier materials but also leverages multiple pharmacological effects to achieve multi-agent combination therapy [25,26,27,28].

Natural polysaccharides are widely used in nanomedicine as immunomodulatory active ingredients. Polysaccharide-based nanostructures not only provide protective matrices for drugs, enhancing their stability and delivery efficiency, but also possess inherent biological activity. *Cordyceps* polysaccharides, regarded as key immunomodulatory constituents of *Cordyceps* sinensis, have attracted considerable interest due to their pronounced immunomodulatory, antitumor, and anti-inflammatory activities [29,30,31]. Research indicates that *Cordyceps* polysaccharides exert immunostimulatory effects by modulating T-cell subsets. In addition, their molecular structures are rich in functional groups such as hydroxyl groups, readily facilitating self-assembly via intermolecular interactions like hydrogen bonding and hydrophobic interactions, thereby providing a structural foundation for carrier-free nanomaterial construction [32,33]. Accordingly, a high drug-loading, low-toxicity nanoscale system formed by combining *Cordyceps* polysaccharides with antitumor drugs exhibits synergistic antitumor effects combining chemotherapy and immunomodulation, demonstrating significant application potential [34,35,36].

**Scheme 1:**
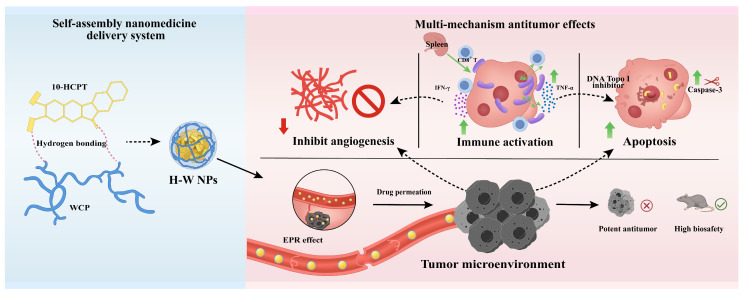
Schematic illustration of the self-assembly nanoscale drug delivery system of H-W NPs (blue panel) and their multi-mechanism antitumor effects (red panel).

Based on the design principle of “Property optimization and Therapeutic synergy”, this study employed the naturally occurring active polysaccharide component and the broad-spectrum chemotherapeutic agent to construct a self-assembling nanoscale drug delivery system (Scheme 1). The system was formed through non-covalent interactions between the two components, including hydrogen bonding and hydrophobic interactions. This nanomaterial system improved the stability and solubility of HCPT through nanoscale modification, thereby optimizing its in vivo delivery and bioavailability while reducing systemic toxicity. In addition, WCP’s immunomodulatory activity acted in concert with HCPT’s cytotoxic effects to achieve dual antitumor activity through “direct chemotherapeutic killing + immune regulation”. We further employed multiple characterization techniques to elucidate the self-assembly mechanism of the nanoparticles. Using melanoma as a disease model, we conducted systematic in vitro and in vivo experiments to evaluate its antitumor efficacy and safety. The results indicated that this strategy enhanced therapeutic efficacy while reducing systemic toxicity, thereby offering a promising nanotherapeutic approach for melanoma treatment.

## 2. Materials and Methods

### 2.1. Materials

10-Hydroxycamptothecin (HCPT) and *Cordyceps* polysaccharides (WCP) were purchased from Shanghai Yuanyi Biotechnology Co., Ltd. (Shanghai, China). Ethanol, acetone, and methanol were all HPLC grade, purchased from Tianjin Damiao Chemical Reagent Factory (Tianjin, China). All other reagents were analytical grade, and purified deionized water was used throughout the experiment.

### 2.2. Animals and Cells

Eight-week-old female C57BL/6 mice were obtained from SPF (Beijing, China) Biotechnology Co., Ltd. All animal experiments were conducted in accordance with the National Research Council’s Guide for the Care and Use of Laboratory Animals and approved by the Experimental Animal Ethics Committee of Beijing University of Chinese Medicine (Certificate No: BUCM-2024120701-4283).

B16-F10 cells and Raw264.7 were both purchased from the China Cell Line Resource (No: 2024101930051) and cultured in RPMI-1640 medium supplemented with 10% FBS, 100 units/mL penicillin, and 100 μg/mL streptomycin at 37 °C in a humidified atmosphere containing 5% CO_2_ (DMEM medium was used for Raw264.7). Prior to experimentation, logarithmic-phase cells with good morphology, uniform adherence, no contamination, and approximately 80% confluence were selected. The spent medium was discarded, and the cells were gently washed with PBS. Then 0.25% trypsin was added to cover the cell layer, and the cells were digested at 37 °C. Digestion was terminated by adding serum-containing RPMI-1640 medium. The cells were gently pipetted to obtain a single-cell suspension (Raw cells require no trypsin digestion; simply gently pipette the adherent cells). They were then collected and centrifuged at 1000 rpm for 5 min. The supernatant was discarded, and the pellet was resuspended in fresh medium. Subsequent processing was performed as required by the experiment.

### 2.3. Preparation and Characterization of H-W NPs

#### 2.3.1. Preparation of H-W NPs

Using the ultrasonic anti-solvent method, WCP was dissolved in water, while HCPT was dissolved in an acetone-ethanol mixture (HCPT:WCP, *w*/*w* = 1:3). HCPT solution was then added dropwise to the aqueous WCP solution under ultrasonication. The mixture was sonicated at 200 W, followed by the removal of organic solvents via rotary evaporation under reduced pressure. The resulting solution was centrifuged at 4000 rpm for 3 min to obtain H-W NPs.

#### 2.3.2. Characterization of H-W NPs

The physicochemical properties and stability of H-W NPs were characterized as follows. Particle size, polydispersity index (PDI), and zeta potential were measured at room temperature using a Zetasizer Nano ZS system (Malvern, UK). Morphology was examined by scanning electron microscopy, and surface elemental composition was analyzed via TEM-EDS. Functional groups of HCPT, WCP, and lyophilized H-W NPs were assessed using Fourier-transform infrared spectroscopy (FTIR). For storage stability, particle size and PDI were monitored over 10 days using a Zetasizer Nano ZS system. Physiological stability was evaluated by incubating H-W NPs in 10% glucose, PBS, or FBS at 37 °C and measuring size and PDI at selected time points.

High-performance liquid chromatography (HPLC) was used to estimate the drug loading (DL) and encapsulation efficiency (EE) of HCPT. The concentration of HCPT was determined using a HPLC system (Thermo, UltiMate 3000, USA), employing methanol-acetonitrile-0.1% acetic acid in water (25:25:50, *v*/*v*/*v*) as the mobile phase. The analysis was performed at 25 °C with a flow rate of 1.0 mL/min and an injection volume of 20 uL. The detection wavelength was set at 320 nm (UV detector, DIONEX, Sunnyvale, CA, USA). This method demonstrated specificity for HCPT and exhibited good linearity (Y = 1.3016X + 0.0729, R^2^ = 0.9993).

In vitro release was studied under different pH conditions (7.4, 6.8, and 5.7). A dialysis bag (MWCO:3000 Da) containing 2 mL of H-W NPs was immersed in 50 mL of PBS with 0.1% Tween 80 and shaken at 200 rpm and 37 °C. At various time points (0 min, 15 min, 30 min, 1 h, 2 h, 4 h, 6 h, 8 h, 10 h, 12 h, 24 h, 36 h, 48 h, and 72 h), 200 μL of dialysate was withdrawn and replaced with an equal volume of fresh release medium. Additionally, the external release solution was replaced every 24 h. The cumulative release of H-W NPs was calculated based on the HCPT content in the release medium. All experiments were performed in triplicate.

### 2.4. Molecular Dynamics Simulation Analysis of H-W NPs

To elucidate the self-assembly mechanism, molecular dynamics simulations were performed. The alkaloid molecules were parameterized using the GAFF force field. The polysaccharide structure, which consists of a glucose backbone with mannose and galactose branches, was modeled in GLYCAM-Web to predict its most probable conformation due to the lack of experimental structural data, and was subsequently parameterized with the GLYCAM-06j force field [32,37,38]. One polysaccharide and six alkaloid molecules were randomly placed in a 7 × 7 × 7 nm^3^ TIP3P water box to construct the initial system. All simulations were conducted using GROMACS 2024.4. Prior to production runs, the system was energy-minimized. Production simulations were performed in the NPT ensemble (100 ns). Resulting trajectories were visualized in VMD 1.9.3.

### 2.5. In Vitro Cytotoxicity Experiments

The cytotoxicity of free HCPT, WCP, and H-W NPs against B16F10 cells was evaluated by MTT assay. Cells (8 × 10^3^ cells/well) were seeded in 96-well plates for 24 h, then treated with serial dilutions of H-W NPs (0.00–10.00 µg/mL), HCPT (0.00–100.00 µg/mL), or WCP (0.00–50.00 mg/mL) for 48 h in six replicates. Following MTT incubation (4 h) and DMSO solubilization, absorbance at 570 nm was measured to determine inhibition rates and IC_50_ values using GraphPad Prism.

### 2.6. In Vitro Cellular Uptake

B16F10 cells in logarithmic growth phase were seeded into 24-well plates at 5 × 10^4^ cells/well and cultured for 24 h. Cells were then treated with HCPT or H-W NPs (both containing 5 µg/mL HCPT) for 1, 3, and 5 h. At each time point, cells were fixed with 4% paraformaldehyde for 20 min, washed twice with PBS, stained with 0.5 µg/mL DAPI for 10 min, and then 300 µL PBS was added. Cellular uptake was visualized under an inverted fluorescence microscope (TY2024004920, Yijingtong Optical Technology Co., Ltd., Guangzhou, China).

### 2.7. To Investigate the Effect of H-W NPs on ROS Secretion and NO Level by Raw Cells In Vitro

Cells were seeded at 5 × 10^5^ cells/mL in 96-well and 6-well plates for 24 h, then treated with LPS (1 µg/mL) alone or combined with WCP (250 µg/mL) or H-W NPs for 24 h. Intracellular ROS was measured by incubating cells with DCFH-DA (10 µg/mL, 200 µL) for 25 min at 37 °C, followed by fluorescence detection (ex/em: 488/525 nm). NO production was assessed by mixing supernatants (50 µL) with Griess reagents and measuring absorbance at 540 nm.

### 2.8. Antitumor Effect of H-W NPs In Vivo

#### 2.8.1. Antitumor Effect In Vivo

Healthy female C57BL/6 mice (20–22 g) were subcutaneously inoculated with B16F10 cells (1 × 10^7^ cells/mL, 0.1 mL) near the right lower limb. When tumor volume reached approximately 50 mm^3^, mice with similar tumor sizes were randomly assigned to five groups (*n* = 6). Each group received tail vein injections every other day for 12 consecutive days. The injection volume per dose was 0.2 mL, with a dosage of 5 mg/kg; all drugs were diluted in 5% glucose solution prior to administration. The treatment groups were:

Negative; HCPT; WCP; HCPT + WCP (H + W); HW NPs (H-W)

Tumor length and width were measured every other day with a digital caliper (0.01 mm accuracy). On day 12, mice were euthanized, and tumors were excised and weighed. Tumor volume and inhibition rate were calculated as:

Volume = (length × width^2^)/2 (1)

Tumor inhibition rate (%) = (1 − Wt/Wn) × 100%(2)

Wt, Wn: tumor weight in the treatment group and the negative control group.

Tumor tissue was fixed in 4% paraformaldehyde, paraffin-embedded, sectioned, and stained with hematoxylin and eosin (H&E) for histological evaluation under light microscopy.

#### 2.8.2. Study on TUNEL Immunofluorescence Staining

Tumor samples were fixed in 4% paraformaldehyde, paraffin-embedded, sectioned, and deparaffinized. After Proteinase K digestion at 37 °C for 30 min, sections were incubated with TUNEL reaction solution (50 μL) at 37 °C for 2 h in darkness, counterstained with DAPI for 10 min, mounted with anti-fade medium, and imaged by fluorescence microscopy.

#### 2.8.3. Immunohistochemistry Investigation of Tumor Tissue and Spleen Tissue

Tumor tissue and spleen tissue were paraffin-embedded, sectioned, and dewaxed. Following antigen retrieval and hydrogen peroxide treatment to block endogenous peroxidase, sections were incubated with primary antibodies (CD31 and caspase-3), then secondary antibodies. After DAB staining and hematoxylin counterstaining, sections were dehydrated, mounted, and examined microscopically. Nuclei appeared blue, while positive DAB expression showed brownish-yellow.

### 2.9. In Vivo Imaging and Quantitative Biodistribution

Tumor targeting and in vivo distribution of H-W NPs in tumor-bearing animals were assessed using the IVIS spectrum imaging system (Hopkinton, MA, USA). B16-F10 tumor-bearing mice were split into two groups of three mice each, and 100 µL of either DiR + HCPT or DiR-HW was injected into their tail veins. DiR was physically mixed with HCPT to create DiR + HCPT, and DiR was conjugated to H-W NPs to create DiR-HW. With excitation at 748 nm and emission at 780 nm, the IVIS spectrum imaging system was used to track tumor accumulation and organ distribution at 1, 3, 6, 9, 12, and 24 h after injection. Living Image^®^ software 4.4 was used to quantify the fluorescence signals. Minor variations in animal posture during imaging were unavoidable and mitigated by quantitative ROI analysis and ex vivo biodistribution. At 24 h post-injection, tumor tissue and major organ tissues (heart, liver, spleen, lung, and kidney) were harvested for imaging analysis. All images were acquired using identical imaging parameters and displayed within the same group using a uniform fluorescence intensity scale.

### 2.10. Antitumor Biosafety of HCPT Based on H-W NPs In Vivo

After the initial dose, the mice were weighed (accurate: 0.1 g) and observed for activity, sleep, and death throughout the treatment period. On day 12, the tumor tissue and major organ tissues (heart, liver, spleen, lung, and kidney) were collected from the mice. The organ indices were calculated according to Formula (3):

CIR (%) = WC/Wm; LIR (%) = WL/Wm; SIR (%) = WS/Wm; LuIR (%) = WLU/Wm; RIR (%) = WR/Wm(3)

WC, WL, WS, WLU, and WR represent the weights of the heart, liver, spleen, lung, and kidney, respectively. Wm denotes the body weight of animals in each group.

Collected organs were fixed in 4% paraformaldehyde for 24 h, dehydrated, paraffin-embedded, sectioned, and stained with hematoxylin and eosin for histopathological evaluation by light microscopy.

### 2.11. Study on Immune Effects In Vivo

#### 2.11.1. Immunofluorescence Staining of CD4^+^ T Cells/CD8^+^ T Cells in Tumor Tissue and Spleen Tissue

Spleen tissue and tumor tissue were fixed, paraffin-embedded, sectioned, and deparaffinized. Following antigen retrieval in EDTA or citrate buffer and peroxidase blocking with 3% H_2_O_2_, sections were incubated with primary antibodies (CD4 or CD8) overnight at 4 °C, then with HRP-conjugated secondary antibodies and tyramide amplification (TYR-570). Nuclei were counterstained with DAPI before fluorescence imaging.

#### 2.11.2. Investigation on the Proportion of Antigen-Specific T Cells in Spleen Tissue

Flow cytometry was employed to quantify CD4^+^ T cell and CD8^+^ T cell populations in mouse spleens. Spleens were dissected and mechanically dissociated through a 70 μm cell strainer to obtain single-cell suspensions. Following centrifugation and resuspension in PBS, red blood cells were lysed for 1 min, and cells were collected by centrifugation at 300× *g* for 10 min. Cell suspensions (1 × 10^6^ cells/100 μL) were stained with Zombie viability dye for 15 min, then incubated with fluorescent antibodies (Mouse CD45 APC-CY7, CD3 BV421, CD4 FITC, and CD8 PE-CY7) for 15 min in darkness. After two PBS washes, cells were resuspended in 200 μL PBS for flow cytometric analysis.

#### 2.11.3. Cytokine Detection

The blood of each group of mice was taken from the canthus of the eyes. The serum was obtained by centrifuging at 4 °C at 3000 rpm for 5 min, and the different cytokines in the serum (TNF-α, IFN-γ, and IL-6) were quantitatively analyzed. Cytokines are detected using the ELISA kit according to the manufacturer’s protocol.

### 2.12. RNA Sequencing Analysis

Total RNA was extracted from tumor tissue across all treatment groups. RNA sequencing was performed on the Illumina NovaSeq 6000 platform (Illumina, Inc., Hayward, CA, USA) according to standard protocols for library concentration and data output. Differentially expressed genes (DEGs) were subjected to functional enrichment studies, such as Gene Ontology (GO) and Kyoto Encyclopedia of Genes and Genomes (KEGG) pathway analysis.

### 2.13. Statistical Analysis

Data were processed using GraphPad Prism 8.0. The data presented were biological replicates from at least three independent experiments, expressed as the mean ± standard deviation (SD) of the measured values. Differences between groups were evaluated using analysis of variance (ANOVA) and Student’s *t*-test.

## 3. Results and Discussion

### 3.1. Characterization of H-W NPs

**Figure 1:**
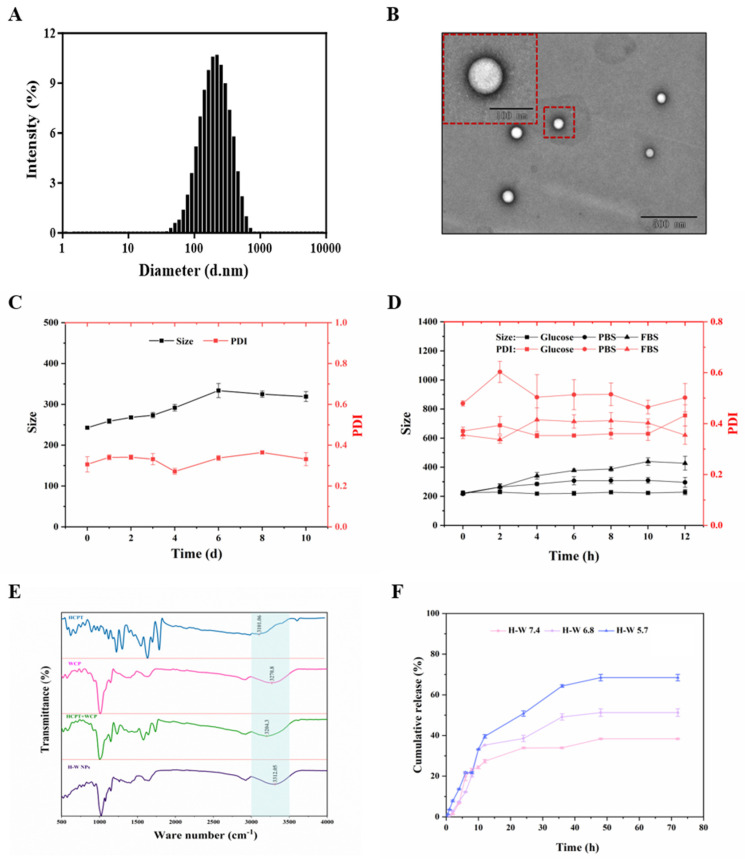
(**A**) Particle sizes of H-W NPs (*n* = 3). (**B**) SEM images. The red-outlined panel shows a higher-magnification SEM image (scale bars: 100 nm and 500 nm). (**C**) Time stability (10 days, *n* = 3). (**D**) Stability in three solution media within 12 h (10% glucose solution, PBS, and FBS, *n* = 3). (**E**) Infrared spectra of HCPT, WCP, HCPT + WCP, and H-W NPs. (**F**) In vitro release profiles of H-W NPs over 72 h in release media at different pH conditions (*n* = 3).

The H-W NPs exhibited an average particle size of 247.13 ± 7.85 nm with a PDI of 0.28 ± 0.01, showing a narrow size distribution (Figure 1A). SEM images revealed nearly spherical, non-aggregated particles without drug crystals (Figure 1B). Surface elemental analysis indicated that H-W NPs exhibited lower nitrogen content compared to HCPT, suggesting that HCPT was encapsulated within the core–shell structure (Table 1). This finding corroborated the aforementioned results. During 10-day storage, particle size remained relatively stable with minimal precipitation, demonstrating good in vitro stability (Figure 1C). The nanoparticles also showed favorable stability in both glucose solution and PBS, with fluctuations in particle size and PDI remaining within acceptable ranges. A slight increase in particle size was observed in FBS, which was likely due to protein adsorption on the nanoparticle surface and subsequent protein corona formation [39]. Despite this increase, no significant aggregation or precipitation was observed within 12 h, indicating that H-W NPs maintained good stability in a blood-mimicking environment. Therefore, glucose solution or PBS was selected as the in vivo delivery medium for subsequent experiments (Figure 1D). FTIR spectra (Figure 1E) showed that the H-W NPs exhibited a broadened hydroxyl absorption peak in the 3000–3500 cm^−1^ region compared with other groups, suggesting that the self-assembly process between HCPT and WCP was influenced by intermolecular hydrogen bonding interactions. The DL was 51.45% ± 0.043% for HCPT. The EE was 61.28% ± 0.12% for HCPT.

In vitro release profiles demonstrated pH-dependent HCPT release, with accelerated rates at lower pH levels (Figure 1F). This pH-responsive behavior enabled slow drug release under physiological conditions (pH 7.4), minimizing premature leakage while achieving rapid release in acidic tumor microenvironments (pH 6.8 and 5.0) for enhanced intratumoral delivery.

### 3.2. Molecular Dynamics Simulation Analysis of H-W NPs

**Figure 2:**
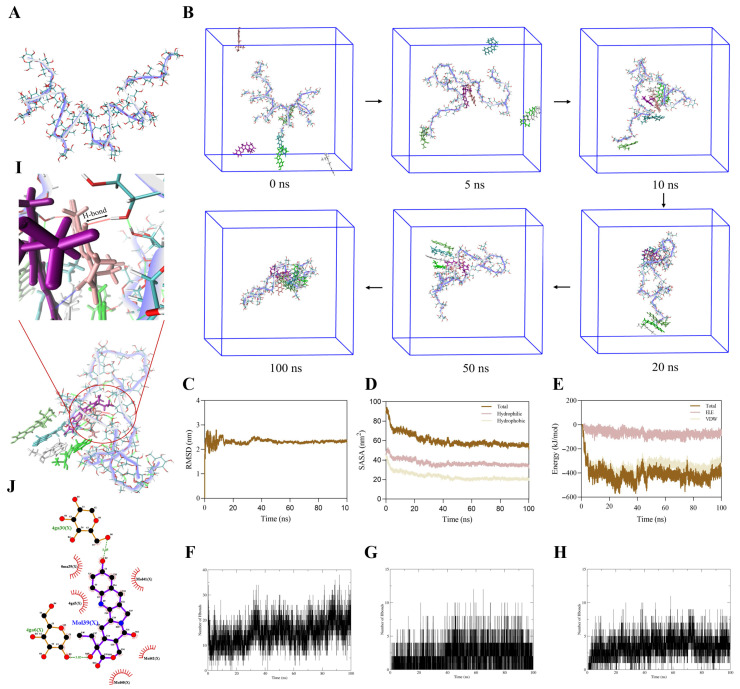
(**A**) Three-dimensional structure diagram of WCP. (**B**) Snapshot of the transient trajectory of the system. (**C**) RMSD calculation. (**D**) SASA calculation. (**E**) Energy calculation. (**F**) Trends in intramolecular hydrogen bonding of WCP. (**G**) Trends in intramolecular hydrogen bonding of HCPT. (**H**) Trends in intramolecular hydrogen bonding of the system. (**I**) Hydrogen bond interaction of the system. The red dashed line denotes a hydrogen bond. (**J**) Two-dimensional interaction of the system.

Molecular-dynamics (MD) simulations indicated that the mannose and galactose branches imposed an initial helical bend on the polysaccharide (Figure 2A). The extended chain progressively attracted and partially enveloped HCPT. After 20 ns, the alkaloid molecules adopted a layered, π-stacked arrangement. Thereafter, the two components spontaneously co-assembled into stable nanoscale aggregates (Figure 2B).

Root-mean-square deviation (RMSD), the cumulative displacement of all atoms relative to the reference structure, is a standard metric of system stability and convergence. A plateau phase commenced after approximately 40 ns (Figure 2C), indicating that the overall system structure had reached a stable state.

Solvent-accessible surface area (SASA) provides a measure of nanoparticle compactness; SASA exhibited a sustained decline throughout the trajectory (Figure 2D) and leveled off at later stages. This progressive reduction in SASA indicated continuous contraction of the self-assembled aggregates, potentially suggesting reduced contact between the hydrophobic core and water. This trend was consistent with the experimentally observed expectation of enhanced dispersion stability after drug nanosizing.

To quantify the driving mechanism, van der Waals (VDW) and electrostatic (ELE) interaction energies, together with hydrogen-bond distributions, were monitored concurrently. Figure 2E reported mean VDW and ELE energies between polysaccharide and alkaloid of −341.75 ± 58.98 kJ mol^−1^ and −68.89 ± 24.60 kJ mol^−1^, respectively, indicating that dispersion interactions dominated the association. Trajectory analysis showed that the number of intramolecular hydrogen bonds within WCP increased gradually (Figure 2F), consistent with enhanced internal stability after alkaloid binding. Following HCPT aggregation, a small number of intermolecular hydrogen bonds emerged (Figure 2G). Intermolecular hydrogen bonds between WCP and HCPT exhibited a similar trend, with occupancies centered at 3–5 bonds (Figure 2H,I). The two-dimensional interaction map (Figure 2J) illustrated one HCPT molecule forming two hydrogen bonds with two distinct monosaccharide units of WCP. These data demonstrated that a stable hydrogen-bond network consolidated the nanoscale assembly [40,41]. Collectively, hydrophobic, π-π stacking, electrostatic, van der Waals, and hydrogen-bonding interactions drove the co-assembly, with van der Waals forces and hydrogen bonds providing the principal energetic terms.

### 3.3. In Vitro Cytotoxicity Experiments

**Figure 3:**
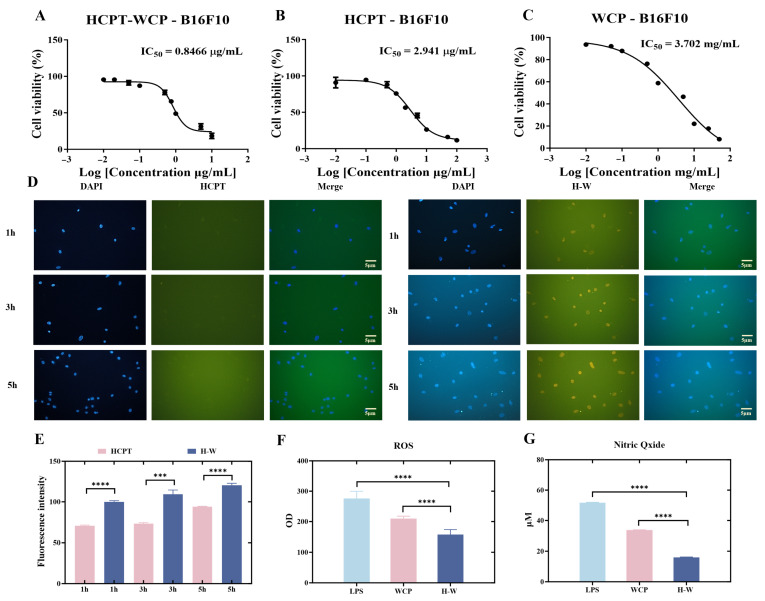
(**A**–**C**) Cytotoxicity assessment of H-W NPs (**A**), HCPT (**B**), and WCP (**C**) on B16-F10 cells (*n* = 3). (**D**) Cell uptake and intracellular distribution of H-W NPs and HCPT (1 h, 3 h, and 5 h). (**E**) Fluorescent semi-quantitative results (*n* = 3). (**F**) Effects of H-W NPs and WCP on ROS levels in RAW cells (*n* = 3). (**G**) Effects of H-W NPs and WCP on NO levels in RAW cells (*n* = 3). Data were shown as mean ± SD. Student’s *t*-test was used for (**E**), and ANOVA was used for (**F**,**G**). *** *p* < 0.001, **** *p* < 0.0001.

As shown in Figure 3A–C, the order of cytotoxicity was H-W NPs > HCPT > WCP. The IC_50_ values for WCP, HCPT, and H-W NPs were 3.702 mg/mL, 2.941 µg/mL, and 0.8466 µg/mL, respectively. The inhibition rate of H-W NPs against B16-F10 cells was threefold higher than that of HCPT, indicating that H-W NPs exhibited a stronger inhibitory activity toward B16-F10 cells and demonstrated a marked pharmacological advantage at the cellular level. This advantage may be related to the synergistic antitumor effects of HCPT and WCP [42,43,44].

### 3.4. In Vitro Cellular Uptake

As shown in Figure 3D,E, over a 5 h period, H-W NPs exhibited significantly higher green fluorescence intensity than the HCPT group, indicating enhanced cellular uptake of H-W NPs by B16-F10 cells. This effect may be attributed to hydrophilic moieties in WCP, which improve HCPT solubility and colloidal dispersion stability, thereby reducing precipitation and increasing the bioavailable drug concentration accessible to tumor cells, which in turn may enhance passive diffusion [45,46]. In addition, the polysaccharide layer on the surface may encourage internalization through endocytosis mediated by clathrin, further contributing to the increased cellular signal [47].

### 3.5. Effect of H-W NPs on ROS and NO Secretion by Raw Cells In Vitro

As shown in Figure 3F,G, the results of the model group indicated that LPS successfully induced elevated levels of ROS and NO within Raw cells. Compared to the control group alone, H-W NPs significantly reduced ROS and NO secretion in Raw cells, indicating superior anti-inflammatory activity in vitro. This effect may be related to the immunomodulatory properties of the WCP in the nanoparticle outer layer, suggesting that the H-W NPs can reduce accumulated ROS and NO in the hypoxic tumor microenvironment, alleviate immune dysfunction caused by high oxidative stress, and modulate systemic antitumor immune responses [48,49].

### 3.6. Enhanced Antitumor Effects of H-W NPs In Vivo

**Figure 4:**
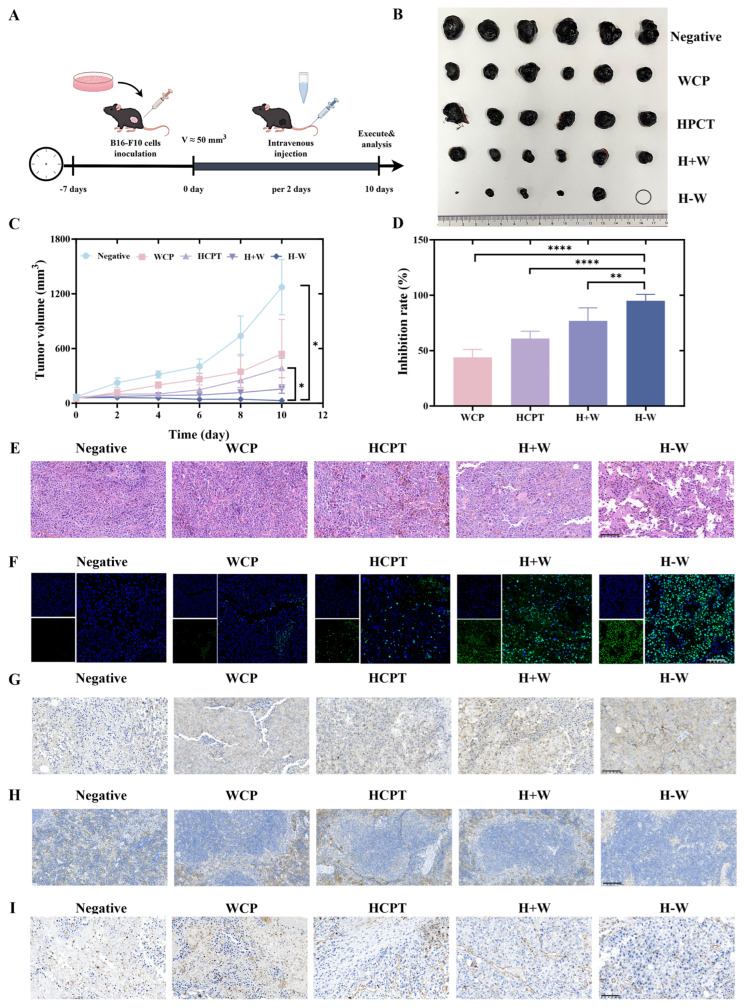
(**A**) Animal experimentation flow chart. A total of six doses were administered on days 0, 2, 4, 6, 8, and 10. (**B**) Representative images of tumors in each group (*n* = 6). The circle indicates that the tumor volume is negligible. (**C**) Tumor volume in each group (*n* = 6). (**D**) Inhibition rate in each group (*n* = 6). (**E**) Histopathology of tumor tissue following treatment. (**F**) TUNEL staining images of tumor tissue in mice. (**G**) The expression of caspase-3 by IHC analysis of the collected tumor tissue. Positive staining appears brownish-yellow. (**H**) The expression of caspase-3 by IHC analysis of the collected spleen tissue. (**I**) The expression of CD31 by IHC analysis of the collected tumor tissue. Positive staining appears brownish-yellow. Data were shown as mean ± SD. ANOVA was used for statistical analysis. * *p* < 0.05, ** *p* < 0.01, **** *p* < 0.0001.

The in vivo antitumor effect of H-W NPs was assessed using B16-F10-tumor-bearing mice as the animal model. As exhibited in Figure 4A–C, Free HCPT, H + W, and H-W NPs slowed the growth rate of tumors compared with the negative control group. As shown in Figure 4D, the inhibition rate was 95.08% for the H-W NPs, much higher than the H + W (95.08% vs. 76.92%) and the HCPT (95.08% vs. 60.85%). As for H-W NPs, the best tumor inhibition effect was achieved during the whole therapeutic period. Furthermore, we used H&E staining to evaluate the tumor necrosis in each treatment group. As shown in Figure 4E, compared to other groups, tumor slices from the H-W NPs group showed a marked scarcity of intact cell nuclei, presenting incomplete and atrophied nuclear morphology, tissue disintegration with distinct fissures, and pink necrotic areas. These results indicated that H-W NPs can disable tumor cells more efficiently.

Apoptosis was assessed using the TUNEL assay (Figure 4F). Compared to other groups, the H-W NPs group exhibited stronger green fluorescence signals, suggesting more pronounced DNA damage. This result suggested that H-W NPs might have had an advantage in inducing apoptosis. Through immunohistochemical (IHC) staining of caspase-3, a key executor protein in apoptosis, this study further evaluated the pro-apoptotic effect of H-W NPs in vivo. As shown in Figure 4G–I, compared with the other groups, caspase-3 positive staining (brownish-yellow areas) in tumor tissues was markedly increased in the H-W NPs group, suggesting that H-W NPs effectively induced tumor cell apoptosis in vivo. Concurrently, the H-W NPs group exhibited reduced CD31 expression levels compared to other groups, suggesting inhibition of tumor angiogenesis. These results collectively demonstrated that the H-W NPs not only promoted tumor cell apoptosis but also suppressed angiogenesis. Additionally, in spleen tissue, caspase-3 expression was downregulated in the H-W NPs group. These findings suggested that H-W NPs may preserve the proliferative and activated capacity of splenic lymphocytes by reducing tumor microenvironment-induced splenic apoptosis, thereby enhancing the body’s antitumor immune responses. Across the aforementioned indicators, the H-W NPs group exhibited superior overall performance compared with both the free-drug group and the H + W group. This therapeutic difference might be attributed to the improved drug-delivery advantages afforded by the nano formulation [50].

### 3.7. In Vivo Imaging and Quantitative Biodistribution

**Figure 5:**
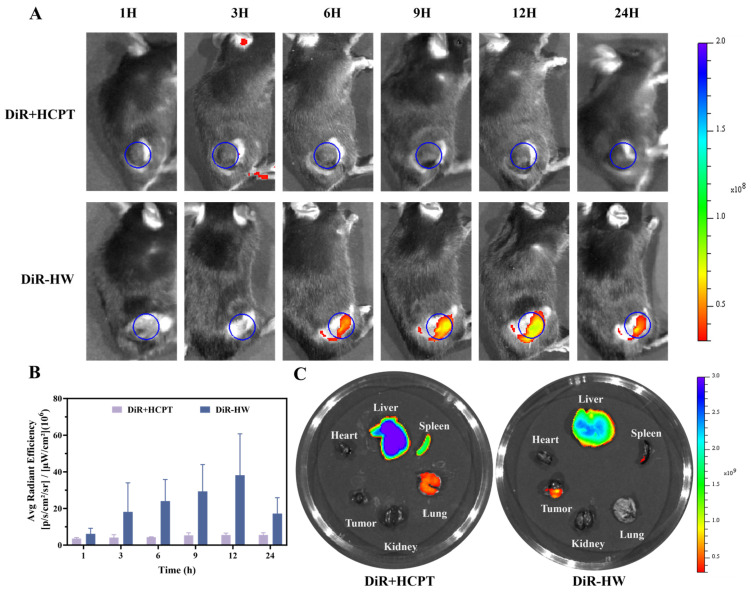
(**A**) In vivo fluorescence imaging of DiR + HCPT, DiR-HW in B16-F10 tumor-bearing mice (*n* = 3). The blue circles denote regions of ROI for quantitative analysis. (**B**) The corresponding quantitative analysis of (**A**). All images were acquired under identical settings, and fluorescence signals were quantified using same-size ROIs placed over the tumor. Data are presented as mean ± SD. (**C**) Ex vivo fluorescence imaging of major organs and tumors at 24 h post-administration of DiR + HCPT and DiR-HW.

To achieve effective antitumor effects, H-W NPs must accumulate and penetrate tumor sites to avoid rapid clearance by the body. As shown in Figure 5A,B, mice in the free DiR + HCPT group consistently displayed a faint fluorescence signal at every time point. In contrast, the fluorescence signal in the DiR-HW group gradually increased after tail vein injection, reaching a peak at 2 h and persisting for up to 24 h. In vitro fluorescence imaging results of major organs and tumors further validated that H-W NPs can effectively accumulate at tumor sites. Tumor tissue from the DiR-HW group mice exhibited significantly stronger fluorescence signal intensity (Figure 5C). These results indicated that the free drug was rapidly cleared in mice with poor tumor enrichment capacity. H-W NPs demonstrated marked accumulation in tumors, suggesting enhanced penetration and retention at tumor sites. This might have been attributable to their negatively charged, hydrophilic surfaces, which likely enhanced blood stability, thereby promoting EPR-related passive accumulation [51,52,53]. Furthermore, when combined with the in vitro cellular uptake experiments, these findings suggested increased drug delivery into tumor cells, thereby enhancing overall antitumor efficacy.

### 3.8. Antitumor Biosafety of H-W NPs In Vivo

**Figure 6:**
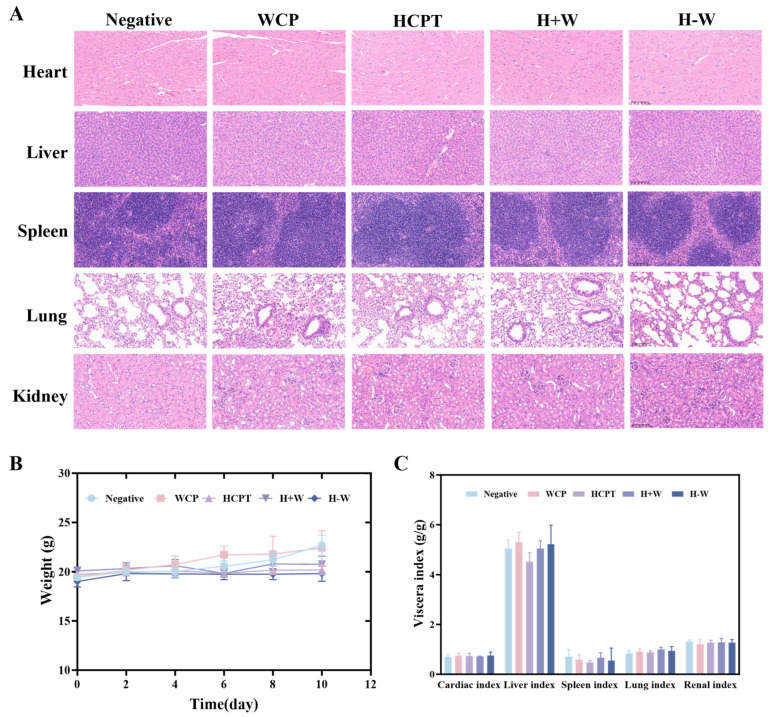
(**A**) Histopathology of major organs in each group after treatment. (**B**) Body weight (*n* = 6). (**C**) Viscera index (*n* = 6).

The biosafety of H-W NPs was assessed using histopathology, body weight, and organ index analysis. The H&E staining of major organ tissues in Figure 6A indicated that all organs in the H-W NPs group retained essentially normal tissue architecture with clearly defined cellular morphology. No obvious pathological changes, including inflammatory cell infiltration, oedema, degeneration, or necrosis, were observed. These findings indicate that H-W NPs exhibit favorable biocompatibility and in vivo safety. Compared to other groups, mice in the H-W NPs group exhibited no significant changes in body weight (Figure 6B), suggesting no drug-induced toxic side effects in this group. Further analysis of organ indices in Figure 6C revealed consistent trends, indicating that H-W NPs did not induce significant systemic toxicity.

### 3.9. In Vivo Antitumor Activity of H-W NPs

**Figure 7:**
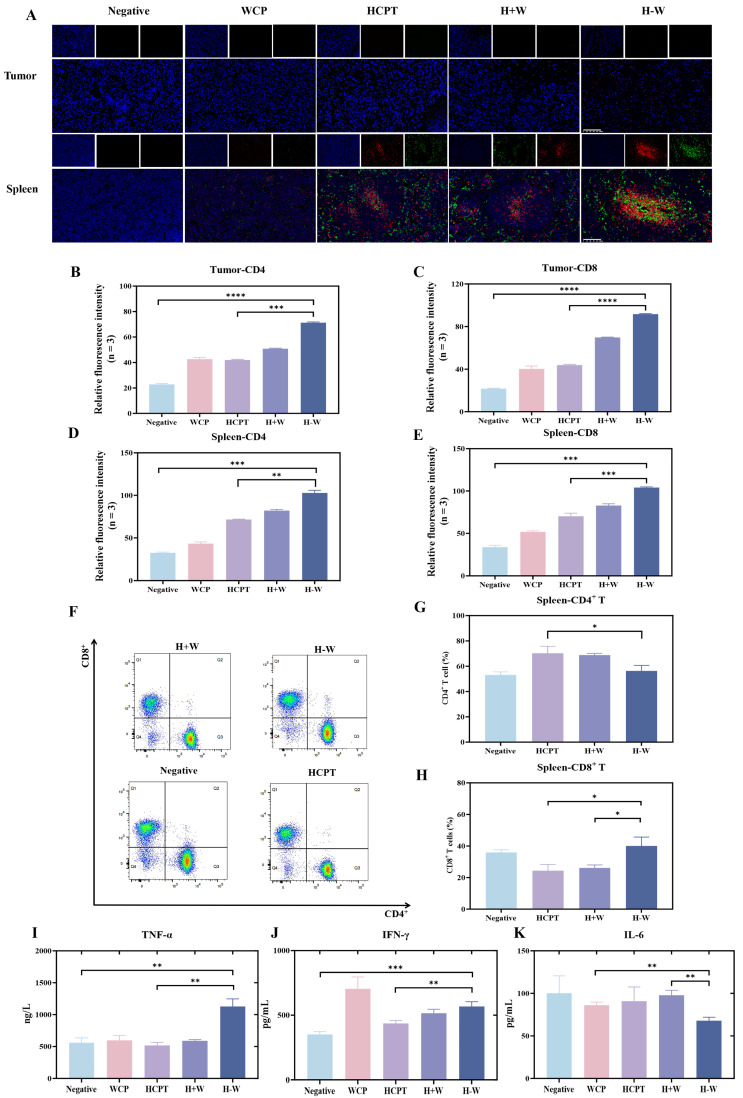
(**A**) Immunofluorescence analysis for CD4^+^ T cells and CD8^+^ T cells in tumor tissue and spleen tissue. (**B**–**E**) Fluorescence semi-quantitative analysis of CD4^+^ T cells and CD8^+^ T cells (*n* = 3). (**F**–**H**) Flow cytometry analysis of the proportion of CD4^+^ T cells and CD8^+^ T cells in spleen tissue (*n* = 3). (**I**–**K**) Quantitative analysis of TNF-α, IFN-γ, and IL-6 levels in the same tumor samples by ELISA (*n* = 3). Data were shown as mean ± SD. ANOVA was used for statistical analysis. * *p* < 0.05, ** *p* < 0.01, *** *p* < 0.001, **** *p* < 0.0001.

We performed immunofluorescence staining to assess the infiltration of CD4^+^ T cells and CD8^+^ T cells in tumor tissue and spleen tissue across different treatment groups, in order to evaluate the impact of nanoparticles on the systemic immune response. As illustrated in Figure 7A–E, in tumor tissue, the semi-quantitative fluorescence intensity of CD4 in the H-W NPs group (71.33 ± 0.54) was significantly higher than that in the HCPT group (44.35 ± 0.09). Similarly, the CD8 fluorescence intensity (91.72 ± 0.48) was also markedly elevated relative to the HCPT group (43.76 ± 0.81). In the spleen, the CD4 fluorescence value (102.91 ± 3.05) was significantly higher than that in the HCPT group (73.26 ± 3.34), and the CD8 value (103.88 ± 0.98) was also notably increased compared to the HCPT group (70.34 ± 3.40). These results collectively demonstrated a significant upregulation of CD4^+^ T cell and CD8^+^ T cell infiltration in both tumor tissue and spleen tissue, demonstrating that the H-W NPs effectively stimulated immune system activation. Subsequently, the proportions of CD4^+^ T cells and CD8^+^ T cells in the spleens of mice treated with H-W NPs were quantified by flow cytometry. The percentage of CD8^+^ T cells reached 40.1%, which was significantly elevated compared with that in the HCPT group (24.4%) and the H + W group (26.1%) (Figure 7F–H). These results suggested that H-W NPs effectively activated systemic cytotoxic CD8^+^ T cells, further demonstrating that the H-W NPs can induce cytotoxic T lymphocyte activation and expansion.

We next measured the serum levels of cytokines TNF-α, IFN-γ, and IL-6 in each group by ELISA (Figure 7I–K). The results revealed that, compared with the negative control group, the H-W NPs group exhibited a significant increase in TNF-α and IFN-γ, along with a decrease in IL-6. These data indicated that H-W NPs can modulate the immune microenvironment. TNF-α (tumor necrosis factor) synergized with IFN-γ (an effector molecule enhancing CD8^+^ T cell function) to establish a pro-inflammatory cytotoxic environment [54,55]. TNF-α induced apoptosis, while IFN-γ possessed anti-angiogenic properties that disrupted tumor vascular structures. Furthermore, the downregulation of IL-6 (a promoter of tumor invasion and metastasis) [56], combined with the synergistic effects of the aforementioned key inflammatory factors, collectively mediated significant antitumor effectiveness.

In summary, H-W NPs enhanced the immune microenvironment and systemically improved immune system activation. This effect may have resulted from the combined action of WCP-driven immunomodulation and HCPT-mediated chemotherapy, which stimulated and amplified CD8^+^ T cell-dominant immune responses. This altered immune environment further induced tumor cell apoptosis and inhibited angiogenesis, thereby demonstrating significant antitumor activity in vivo.

### 3.10. RNA Sequencing Analysis of H-W NPs

**Figure 8:**
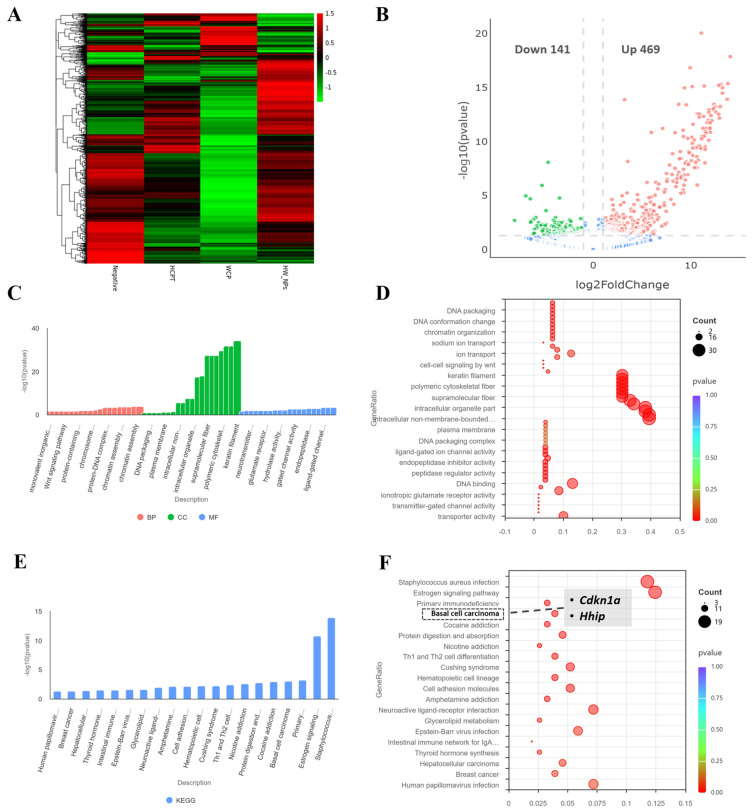
(**A**) Heatmap displaying hierarchical clustering analysis of DEGs between the H-W NPs group and the negative control group. (**B**) Volcano plot showing differentially expressed genes (|log2Fold change| > 1, *p*-value < 0.05). (**C**,**D**) GO functional enrichment analysis of differentially expressed genes. (**E**,**F**) KEGG pathway enrichment analysis of differentially expressed genes (*n* = 3).

RNA sequencing provided deeper insights into the antitumor mechanism of H-W NPs in a melanoma mouse model. Figure 8A showed distinct clustering between H-W NPs and negative control groups, with volcano plot analysis identifying 610 differentially expressed genes (Figure 8B). GO enrichment analysis revealed that H-W NPs primarily impacted DNA structural stability, genetic regulation, and cellular ion homeostasis (Figure 8C,D).

KEGG pathway enrichment analysis identified significant enrichment in cancer pathways, including hepatocellular carcinoma and breast cancer (Figure 8E,F). This confirmed that HCPT, as a TOP I inhibitor, exerted broad-spectrum antitumor activity by inhibiting DNA replication [57]. Notably, enrichment results indicated significant modulation of the Basal cell carcinoma signaling pathway, which showed increased expression of *Cdkn1a* (encoding p21) and *Hhip* (a specific antagonist of the Hedgehog pathway). This expression pattern revealed a synergistic antitumor mechanism: on one hand, the upregulation of p21 blocked the cell cycle, effectively inhibiting cell proliferation [58,59]; on the other hand, the upregulation of *Hhip* disrupted Hedgehog survival signaling, decreasing tumor cell activity and increasing the sensitivity of arrested cells to apoptosis [60,61]. More importantly, inhibition of the Hedgehog pathway released its transcriptional repression of p21, thereby further amplifying the upregulation of p21 [62]. This synergistic mechanism provided explanations at a molecular level for the higher TUNEL positivity rate and enhanced caspase-3 immunofluorescence observed in in vivo experiments. In summary, this study proposed that H-W NPs exerted significant antitumor effects by regulating a dual mechanism of “cell cycle arrest and apoptosis induction”, thereby suppressing cell proliferation and inducing apoptosis.

## 4. Conclusions

This study applied a “Property Optimization and Therapeutic Synergy” design strategy, innovatively incorporating natural active polysaccharides with antitumor chemotherapeutic agents. Through intermolecular interactions enabling self-assembly, a highly stable and efficient antitumor nanoscale drug system (H-W NPs) was successfully constructed. The self-assembled nanostructure significantly enhanced antitumor efficacy while successfully lowering systemic toxicity, as demonstrated by several in vitro and in vivo experiments. The synergistic interaction between the two active components efficiently induced apoptosis and inhibited tumor growth in vivo. Furthermore, the nanoparticles improved the tumor microenvironment and activated antitumor immune responses, thereby achieving dual synergistic therapy through direct drug-mediated killing and immune modulation. In summary, the construction of self-assembled nanoparticles based on green active polysaccharides and chemotherapeutic drugs opens a new avenue for natural polysaccharide-based nanoscale drugs and offers a strategy for the clinical application of chemotherapeutic agents.

## Figures and Tables

**Table 1:** Surface elemental analysis of H-W NPs.

Elements	HCPT (%)	H-W NPs (%)
C	79.9	91.3
O	18.5	7.9
N	1.6	0.8

## Data Availability

The original contributions presented in this study are included in the article. Further inquiries can be directed to the corresponding author.
